# Editorial: Non-invasive Technology Advances in Oncology

**DOI:** 10.3389/fdgth.2021.676216

**Published:** 2021-04-27

**Authors:** Chong Boon Teo, Benjamin Kye Jyn Tan, Dearbhaile Catherine Collins

**Affiliations:** ^1^Yong Loo Lin School of Medicine, National University of Singapore, Singapore, Singapore; ^2^Department of Medical Oncology, Cork University Hospital, Cork, Ireland; ^3^Cancer Research at UCC, College of Medicine and Health, University College Cork, Cork, Ireland

**Keywords:** technology, liquid biopsy and circulating tumor DNA, web-based health information, cryocompression, oncology

Breakthrough non-invasive technologies are swiftly being incorporated in oncology clinical care and research. Broadly, the innovations in this field can be classified by their role(s) in disease and patient management: (1) diagnosis, monitoring, and prognostication, (2) treatment and supportive care, (3) patient self-management and education. In this Research Topic titled “Non-Invasive Technology Advances in Oncology” in Frontiers in Digital Health, we have included recent contributions to this area of research.

Aided by ongoing progress in genomic and molecular methods (1), liquid biopsy is rapidly emerging as one of the kingpins of non-invasive diagnostics and prognostics in oncology. Openshaw and McVeigh comprehensively review the emerging roles of tumor-derived components including circulating tumor cells (CTCs), circulating tumor DNA (ctDNA) and cell-free DNA (cfDNA), messenger and microRNAs, and extracellular vesicles (EVs) in ovarian and endometrial cancer. Compared to conventional tumor biopsies, liquid biopsies offer a non-invasive, high-frequency method of assessing inter- and intra-tumoral heterogeneity to detect, prognosticate and treat cancer. Regarding cancer screening, the authors highlight the absence of a non-invasive screening test with proven survival benefit for patients at risk of ovarian and endometrial cancer, in notable contrast to cervical cancer where Papanicolaou (Pap) smears and human papillomavirus (HPV) tests are routine. Liquid biopsies can supplement existing techniques in detecting ovarian and endometrial cancer, by detecting ctDNA alongside conventional blood biomarkers (CancerSEEK) to improve sensitivity (2), or by detecting ctDNA and exosomes in Pap smear samples routinely taken for cervical cancer screening (PapSEEK) (3). As for disease prognostication, the detection of CTCs in apparently optimally debulked ovarian cancer patients has been shown to predict disease mortality (4), while the presence of ctDNA post-operatively predicts tumor relapse (5–7). Liquid biopsies are useful even for treatment selection, with studies showing that cfDNA BRCA reversion mutations can predict resistance to platinum-based chemotherapy and Poly ADP-ribose Polymerase (PARP) inhibitors. Interestingly, the authors also noted a relative scarcity of evidence on specific liquid biopsy components, such as cell free messenger RNAs (cfmRNA) and cell free microRNAs (cf.miRNA). This highlights the potential for further translational research in liquid biopsy, and on a broader front, in non-invasive technology for cancer diagnostics and prognostics.

In the realm of cancer therapeutics, non-invasive technologies are being harnessed for disease treatment and supportive care. Scalp cooling, now established as a safe and efficacious method of preventing chemotherapy-induced alopecia (8), is one example of a successful non-invasive intervention in supportive care. Efforts in preventing chemotherapy-related adverse effects have now turned to chemotherapy-induced peripheral neuropathy (CIPN), which is a common, dose-limiting and potentially irreversible side-effect of taxane, platinum, and vinca alkaloid-based chemotherapy. Existing interventions for CIPN prevention such as neuroprotective agents and acupuncture have not been proven effective (9–11). Cryotherapy-induced vasoconstriction is an alternative promising non-invasive approach to limit peripheral exposure to toxic chemotherapeutics. While recent landmark trials have supported the use of frozen gloves in preventing CIPN (12, 13), the frozen gloves used in the earlier trial were later recalled due to frostbite and other issues with patient safety and tolerability (14). Here, Binder et al. report a novel mixed-methods approach in the design and development of a limb cryocompression system for CIPN prevention. They identified limb cryocompression (continuous-flow cooling combined with cyclic compression) as a potential alternative to frozen gloves for preventing CIPN, based on early clinical studies that have demonstrated safety, tolerability and potential efficacy (15–17). In designing their protective device, the authors adopted a collaborative approach, which facilitates the integration of evidence-based and user-centered techniques to meet the unique challenges of cross-sector medical device development. This novel approach, named HudPAX, integrates existing design and development methods to overcome the major challenges faced in medical product development such as industrial commercial awareness, medical regulatory standards and market considerations. Today's medical device development endeavors are often best accomplished in a multidisciplinary setting to allow design teams to capitalize on the respective expertise and forte of the clinical, academic, and industry sectors. Centered on cross-sector partnership, the proposed HudPAX approach is highly suited to today's medical innovation climate and can potentially be applied to streamline the development of various other non-invasive technologies for cancer care and beyond.

Apart from the traditional fields of diagnostics and therapeutics, the use of non-invasive digital technology in patient self-management and education is gaining traction. With the increasing pervasiveness of the Internet, online health information, and services are becoming essential for patient self-management in oncology. Despite the burgeoning amount of web-based healthcare information, the probability of accessing health-related information without frustration has not changed significantly over the last decade (18). Hanai et al. thus sought to understand the levels of satisfaction and the factors predicting satisfaction in a cross-sectional study of 412 cancer survivors who used web-based healthcare content. Participants reported using these resources to either record their health or therapy status, relieve their anxiety or stress, or to devise activities of daily living during cancer therapy. Among the 238 participants who used web content to relieve their anxiety or stress, interactive web content (such as social networking services or private blogs, where users had to respond in some form) was associated with significantly increased satisfaction. The authors found no relationship between clinical or demographic factors with the level of satisfaction with web content. Additionally, none of the respondents used evidence-based web healthcare applications backed by clinical trial data. With existing evidence suggesting beneficial effects of self-management interventions on physical and emotional distress and quality of life in cancer patients (19–22), we can expect further progress on the use of internet-enabled technologies to improve holistic care and patient empowerment in oncology. We particularly await the results of randomized trials using wearable technology for patient self-monitoring and for encouraging physical activity in cancer patients (23).

We look forward to receiving the results of additional research in the above fields, as well as in the other aspects that have yet to be covered in this Research Topic. We hope the reader will find this collection a relevant resource that showcases the frontiers of non-invasive technology in oncology.

## Author Contributions

CT and BT wrote the editorial. DC undertook editorial revision and final approval. All authors contributed to the article and approved the submitted version.

## Conflict of Interest

The authors declare that the research was conducted in the absence of any commercial or financial relationships that could be construed as a potential conflict of interest.
